# Supplementary material for: Characterization of Three-Dimensional Trophoblast Spheroids: An Alternative Model to Study the Physiological Properties of the Placental Unit
Source: Cells. 2022 Sep 15;11(18):2884. doi: 10.3390/cells11182884 (PMC9497053; doi:10.3390/cells11182884)
Supplement: Supplementary file 1 [file cells-11-02884-s001.zip › cells-1923845-supplementary.pdf]

cells

MDPI

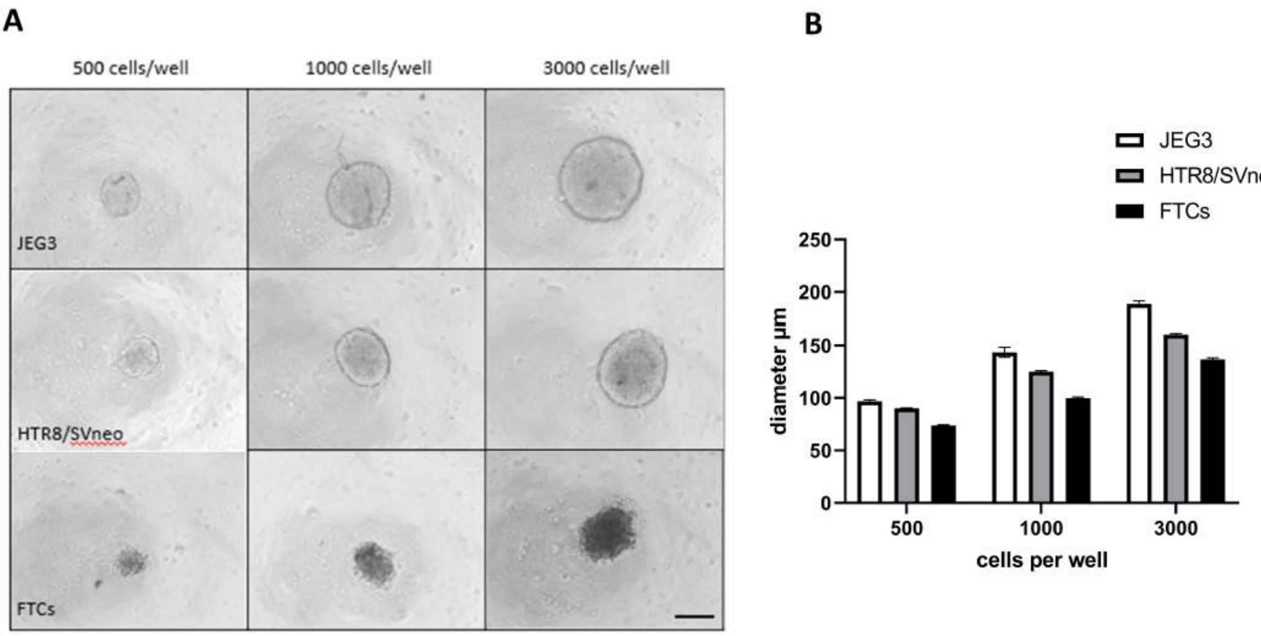

Figure S1. Cell seeding densities establishment for trophoblast spheroids. Bright-field images of JEG3, HTR8/SVneo and FTCs spheroids, scale bar 100 µm (A) and growth size in function of diameter after 24 hours post-seeding (B).
